# Developing and validating an isotrigon texture discrimination task using Amazon Mechanical Turk

**DOI:** 10.1186/1471-2202-16-S1-P278

**Published:** 2015-12-18

**Authors:** John WG Seamons, Marconi S Barbosa, Jonathan D Victor, Dominique Coy, Ted Maddess

**Affiliations:** 1 Eccles Institute for Neuroscience, John Curtin School of Medical Research, ANU, Canberra, ACT 0200, Australia; 2 Department of Neurology & Neuroscience, Weill Cornell Medical College, 1300 York Ave, New York 10021, USA

## 

The human visual system must employ mechanisms to minimize informational redundancy whilst maintaining that which is behaviorally relevant [1,2]. Previous research has concentrated on two-point correlations via spatial frequency and orientation tuning. Higher-order correlations are less studied, but they may inform us about cortical functioning [3]. Isotrigon textures can be used to probe the sensitivity of the human visual system as their structure is exclusively due to 4^th ^and higher-order spatial correlations [4]. Although artificially generated, the same features that give isotrigons salience also create salience in natural images [2]. We implemented an isotrigon discrimination task using the crowdsourcing platform Amazon Mechanical Turk (mTurk) [5]. An important secondary aim was to evaluate the suitability of mTurk for visual psychometric studies as very few exist [6].

**Figure 1:**
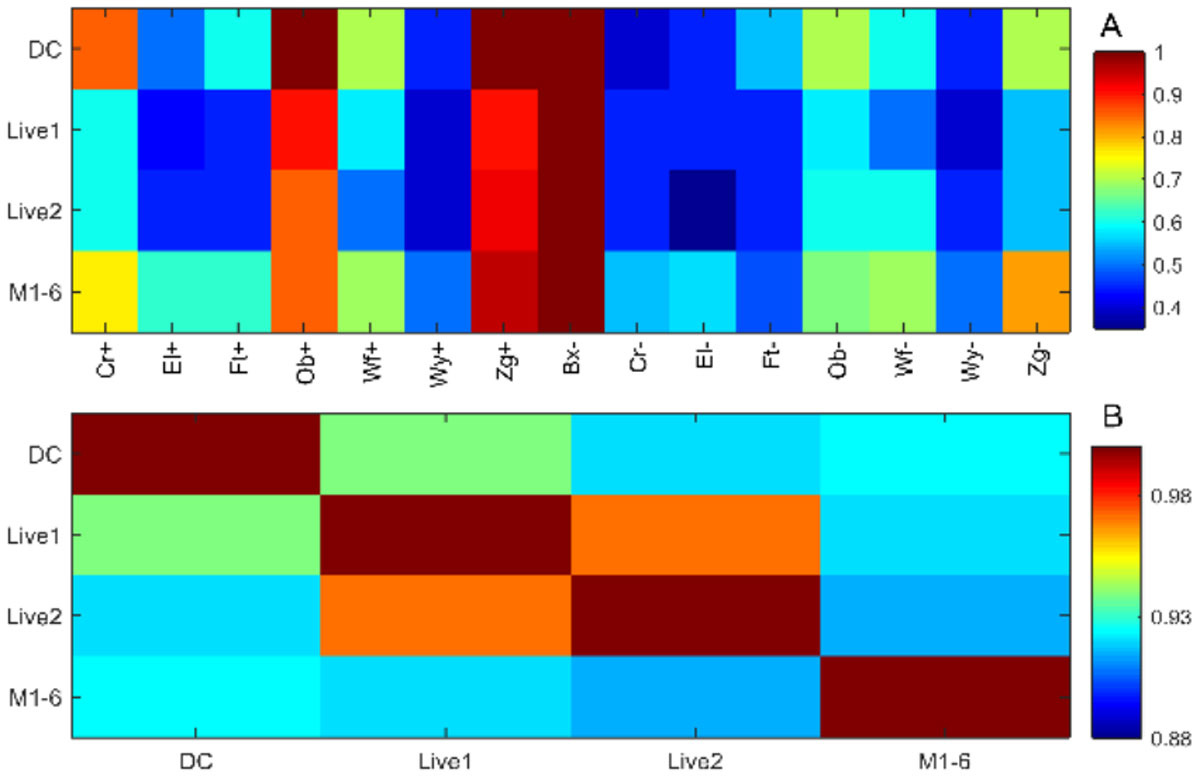
**1A: Color map of median texture discrimination performance versus isotrigon texture type**. Lab datasets DC (84 HITs) and M1-6 (270 HITs). mTurk datasets Live1 (480 HITs) and Live2 (480 HITs). **1B: **Pearson's correlation coefficients between datasets (abbreviations as above).

960 HITs were uploaded to mTurk and 121 naïve subjects participated. Based on data quality, 91% of HITs were retained at a cost of $0.132 AUD per HIT. The mTurk data was compared to two supervised lab datasets. Lab and mTurk performance functions were very similar (Figure 1A) and highly correlated (Figure 1B). Bland-Altman plots were examined and the mean lab/mTurk coefficient of repeatability was 15.5%. Factor analysis was performed on the combined data and 2 principal factors were identified. Previous studies support that the number of mechanisms is less than 10 [7] and more likely 2-4 [8,9]. The congruence between the lab and mTurk data is striking considering the unsupervised mode of delivery. In conclusion, mTurk is an underutilized platform for visual psychometric research which can produce data of comparable quality to lab samples at reduced cost and increased scale.
